# The Association Between Serum Alpha-Carotene and Root Caries in U.S. Adults: A Cross-Sectional Study

**DOI:** 10.3390/life15081188

**Published:** 2025-07-26

**Authors:** Michelle Zak, Yash Brahmbhatt, Abdullah Muhsain, Balqais AlShammari, Badriyah Mandani, Meshari Alenezi, Abdulrahman Salem, Hend Alqaderi

**Affiliations:** 1 Tufts University School of Dental Medicine, Boston, MA 02111, USA; yash.brahmbhatt@tufts.edu; 2 Kuwait Ministry of Health, Kuwait City 15462, Kuwait; drabdullahmuhsain@gmail.com (A.M.); balqeests@hotmail.com (B.A.); b.mandani92@gmail.com (B.M.); meshariii1997@gmail.com (M.A.); abdulrahmansumait@gmail.com (A.S.); 3 Department of Public Health, Tufts University School of Dental Medicine, Boston, MA 02111, USA; hend.alqaderi@tufts.edu; 4 Dasman Diabetes Institute, Kuwait City 15462, Kuwait

**Keywords:** alpha-carotene, root caries, oral health, antioxidant, nutritional intervention, vitamin A precursor

## Abstract

Root caries is a form of decay affecting root surfaces of teeth, often exacerbated by periodontal disease, reduced salivary flow, and compromised mucosal health, all factors strongly influenced by nutrition. Despite this connection, few studies have addressed the role of vitamins in oral health. This study examines the association between serum levels of alpha-carotene (α-carotene), a potent antioxidant and proxy for vitamin A, and the experience of root caries. Using data from the 2017–2018 National Health and Nutrition Examination Survey (NHANES), we conducted a cross-sectional analysis and applied a weighted multiple logistic regression model, adjusting for potential confounders including age, sex, education level, race, income-to-poverty ratio, and presence of gum disease. Higher serum α-carotene levels were inversely associated with root caries. Each unit increase in serum α-carotene was associated with a 9% decrease in the odds of having root caries (OR = 0.91; 95% CI: 0.86–0.97; *p* = 0.004). In this nationally representative sample of U.S. adults, lower serum α-carotene levels were associated with a higher prevalence of root caries. These findings highlight the potential role of nutritional assessment and integration in oral health. Longitudinal and mechanistic studies are needed to confirm and further explore α-carotene’s effects on oral health.

## 1. Introduction

Root caries is a multifactorial, biofilm-mediated dental condition that affects exposed root surfaces, especially among individuals with periodontitis, aggressive toothbrushing habits, and tobacco use [1,2,3]. The cementum covering these surfaces is more porous than coronal enamel and demineralizes at a higher pH, increasing the likelihood of acid-induced demineralization and bacterial invasion [4]. Compared to coronal caries, root caries progresses more aggressively, poses a higher risk of tooth loss, and is more challenging and costly to restore [5,6]. Epidemiological studies show that root caries disproportionately affect older adults due to increased gingival recession, reduced salivary flow, chronic illness, and functional limitations [7,8,9]. According to the World Health Organization, the global population aged 65 and older is expected to double by 2030, which may contribute to a parallel rise in root caries incidence [10]. As older adults retain more natural teeth, the burden of root caries is projected to increase, emphasizing the need for effective and accessible preventive strategies [11].

Key risk factors for root caries development include poor oral hygiene, xerostomia, and nutritional deficiencies [8,12,13,14]. Among these modifiable factors, nutrition has emerged as a promising target due to its role in modulating oxidative stress [15]. Oxidative stress occurs when the production of reactive oxygen species (ROS) exceeds the body’s antioxidant defense capacity, leading to accelerated cellular and tissue damage. In the periodontium, oxidative stress increases the susceptibility to biofilm accumulation and demineralization on root surfaces. It also activates matrix metalloproteinases (MMPs), which degrade the dentin collagen matrix and promote lesion progression [16]. Antioxidants that counteract ROS may mitigate these destructive processes, modulate inflammation, and help preserve periodontal support, limiting conditions that favor root caries formation [17].

Dietary carotenoids are a class of potent antioxidants that may offer oral health benefits [18]. Alpha-carotene (α-carotene) and beta-carotene (β-carotene), abundant in orange, yellow, and dark green vegetables, are classified as provitamin A carotenoids [19]. These compounds are precursors to vitamin A and play essential roles in immune function, epithelial health, and oxidative stress regulation. Vitamin A, a fat-soluble nutrient, is essential for epithelial maintenance, salivary gland function, and immune defense, which are critical mechanisms that support oral health [20,21]. α-carotene has gained attention for its strong antioxidant and anti-inflammatory properties, greater bioavailability, and lower toxicity profile compared to other carotenoids [22,23,24].

While α-carotene concentrations have been widely studied in systemic diseases such as cardiovascular and neurodegenerative disorders [25,26], their role in oral conditions like root caries remains underexplored. The existing literature supports the protective role of carotenoids in periodontal health and underscores the relevance of dietary antioxidants in preserving oral tissues [27,28,29]. For example, a longitudinal study among older Japanese adults found that increased carotenoid intake and elevated serum α-carotene levels were associated with reduced prevalence and severity of periodontal disease [30]. These systemic effects, including reduced oxidative stress, modulation of immune responses, and support for epithelial integrity, may foster a more favorable oral environment, providing biological plausibility for an association with root caries.

The aim of this study was to investigate the association between serum α-carotene levels and root caries experience in a nationally representative sample of U.S. adults using 2017–2018 National Health and Nutrition Examination Survey (NHANES) data. Given α-carotene’s role in supporting antioxidant, immune, and epithelial function, we hypothesize that higher serum α-carotene levels would be associated with a lower prevalence of root caries.

## 2. Materials and Methods

### 2.1. Study Population

This cross-sectional study was conducted using publicly available data from the 2017–2018 National Health and Nutrition Examination Survey (NHANES), a nationally representative survey that evaluates the health and nutritional status of the U.S. civilian, non-institutionalized population across all ages. The survey integrates self-reported data, clinical examinations, and laboratory assessments to ensure a comprehensive dataset that includes demographic information, health indicators, and other variables collected during household interviews. The NHANES program uses a complex, multistage, stratified, and clustered sampling design, ensuring the sample’s representativeness in strict adherence to the protocols upheld by the Centers for Disease Control and Prevention (CDC), Atlanta, GA, USA [31].

The inclusion criteria for the present analysis were subjects from a sample population from the 2017–2018 National Health and Nutrition Examination Survey (NHANES) database who had complete data for both dental examinations and serum α-carotene levels obtained through blood laboratory analysis. Participants without data for either component were excluded from the final sample. The exclusion criteria also included participants with incomplete or missing records to allow for a thorough analysis by ensuring that all participants have the relevant health information to support reliable comparisons. The NHANES program received approval from the National Center for Health Statistics (NCHS) Ethics Review Board, with all participants providing informed consent. This study was exempt from IRB review on 22 January 2025, as it involved the analysis of de-identified, publicly available data and was classified as non-human subject research.

### 2.2. Definition of the Dependent Variable: Root Caries

The primary outcome variable was the presence of root caries, categorized into binary outcomes for statistical analysis: (1) detection of root caries and (2) absence of root caries utilizing “whole mouth” evaluations. Third molars were excluded from the evaluation of root caries. Thus, a maximum of 28 permanent teeth were examined in each participant. Clinical identification of root caries in this study was based on visual and tactile evidence of lesions beginning below the cementoenamel junction (CEJ), particularly in interproximal and facial areas, consistent with established diagnostic patterns [32,33]. As defined by the NHANES database, some visual indicators include color, contour, surface cavitation, and tactile sensation using an explorer, revealing the surface texture. Active root caries appear yellow to brown, while older lesions are darker and harder [34]. These descriptors can be accurately used for the correct diagnosis of root caries, but usually not until the lesion is at an advanced stage. If there is no recession, it is scored as no root caries present. Although it is possible to develop root caries in deep periodontal pockets, there is a very low likelihood of detecting the lesions due to a lack of access and risk of bleeding. Therefore, subgingival examination was not considered.

### 2.3. Description of Independent Variable: α-Carotene Serum Levels (µg/dL)

The primary exposure variable in this study was serum α-carotene level, measured in micrograms per deciliter (µg/dL). Blood samples were collected via venipuncture and analyzed as part of the NHANES laboratory data. α-carotene, a provitamin A carotenoid, serves as a reliable proxy for vitamin A status due to its ability to convert into active vitamin A in the body. Serum α-carotene levels were measured using samples with a target volume of 500 µL to allow for adequate material and potential repeat testing. A carotenoid mixed standard, containing α-carotene, zeaxanthin, and β-cryptoxanthin, was used for quantification. Samples were processed using High-Performance Liquid Chromatography (HPLC), conducted at the Division of Laboratory Sciences, National Center for Environmental Health, CDC, Atlanta, GA, USA. The procedure involved alcohol-based extraction, drying, reconstitution, filtration, and final analysis [35]. Concentrations were calculated against a standard reference and adjusted for consistency using an internal standard. In the dataset, this variable is coded as LBXALC.

### 2.4. Confounding Variables

Confounding variables included demographic and socioeconomic factors such as sex, age, education level, race, the family income-to-federal poverty level (FPL) ratio, serving as a proxy for socioeconomic status, and the presence of gum disease [33,36]. These were adjusted for in the statistical analyses to isolate the effect of α-carotene on the incidence of root caries. A breakdown of the variables can be seen below:

Sex: This was a binary variable, denoted as Males and Females.

Age: This was a continuous variable, depicting age at screening.

Education: Options included Grade Level 0–11, High School/General Educational Development (GED), and education beyond high school.

Race: Participants were asked to identify with a category; Mexican American, White, Black, Asian, or Others.

Ratio of Family Income to Federal Poverty Level: This variable was used to depict economic status relative to the federally defined poverty line. The categories were as follows: <100% FPL, 100–199% FPL, 200–399% FPL, 4000%+ FPL

Presence of Gum Disease: This was a binary variable, where participants were asked, “Have you ever had treatment for gum disease such as scaling and root planning, sometimes called “deep cleaning”?” where (1) represented Yes and (2) represented No.

### 2.5. Statistical Method

Descriptive statistics were calculated to summarize the study population, including weighted percentages for categorical variables and means for continuous variables. Independent sample *t*-tests were used to compare continuous variables between groups. To examine the association between serum α-carotene levels and the presence of root caries, a weighted multivariable binary logistic regression model was applied, adjusting for potentially confounding variables such as sex, age, education, race, income, and presence of gum disease. Statistical significance was set at *p* < 0.05. All analyses were performed using a software called STATA version 17.0 (StataCorp LLC, College Station, TX, USA), incorporating the appropriate sampling weights, strata, and primary sampling units provided by NHANES to account for its complex, multistage sampling design to ensure national representativeness of the U.S. population.

## 3. Results

Table 1 provides a descriptive categorical summary using the NHANES 2017–2018 dataset, focusing on the relationship between serum α-carotene levels and root caries. The study included 19,221 participants, distinguishing those with (N = 1520, 7.91%) and without root caries (N = 17,701, 92.09%).

Table 2 provides a descriptive summary of the continuous variables in comparison with and without root caries. Individuals with root caries exhibited a lower mean serum α-carotene level (3.21 μg/dL) compared to those without root caries (5.17 μg/dL), with a statistically significant difference between the two groups (*p* < 0.0001).

Table 3 shows a weighted multiple logistic regression model depicting the association between participants with root caries and without root caries. For each one-point increase in serum α-carotene level (μg/dL), the odds of having root caries decreased by 9% (OR = 0.91, 95% CI: 0.86–0.97, *p* = 0.004), indicating a significant inverse association.

## 4. Discussion

This nationally representative study of U.S. adults found that lower serum α-carotene levels (μg/dL) were significantly associated with a greater root caries experience.

Root caries typically develops on exposed cementum following gingival recession, where acid erosion and bacterial plaque accumulation frequently occur on root surfaces [37,38,39,40]. Cariogenic bacteria such as *Streptococcus mutans*, *Lactobacillus*, and *Actinomyces* metabolize dietary sugars via glycolysis into organic acids like lactic and acetic acid, lowering plaque pH and promoting mineral loss [41,42]. Root tissues have a relatively high critical pH for demineralization, with cementum at 6.2–6.4 and dentin at around 6.7, making them more prone to acid-induced breakdown [1,13,43]. Prolonged acid exposure degrades mineralized tissues, exposing the collagen matrix, while compromised epithelial barriers may facilitate bacterial infiltration, accelerating lesion progression. If left unmanaged, the progressive combined degradation of mineral and organic components can lead to extensive structural damage and eventual tooth loss [44]. Maintaining gingival tissue integrity is essential, as healthy gingiva serves as a barrier that shields the underlying root surfaces, protecting them from bacterial biofilm [45].

**Figure 1:**
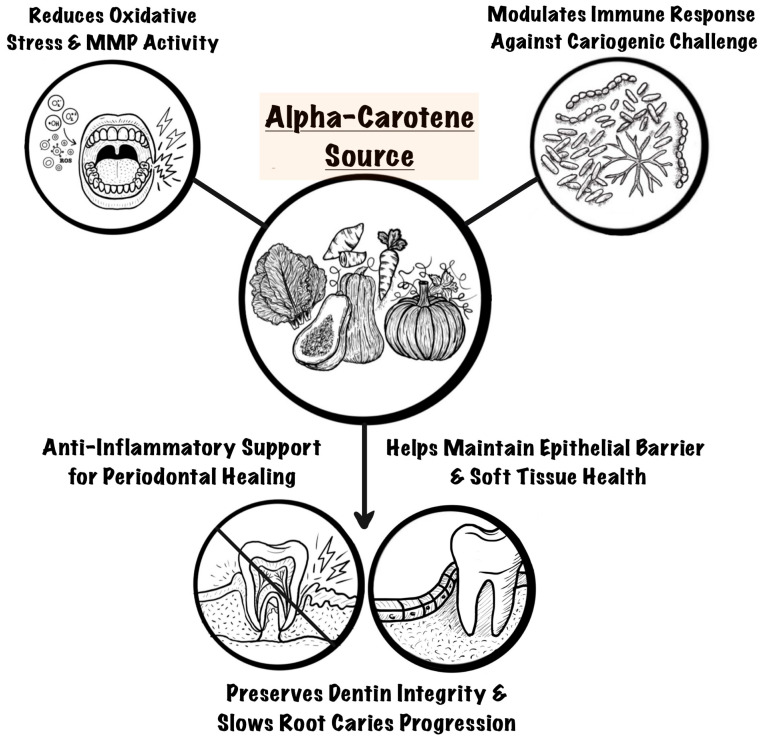
Proposed biological mechanisms by which alpha-carotene may support stabilization of root caries.

α-carotene, a potent dietary antioxidant, may influence root caries progression through multiple mechanisms involving both host and microbial pathways [18,46]. First, it may reduce oxidative stress by neutralizing ROS, which are associated with tissue damage and increased expression of MMPs that degrade the dentin collagen matrix [47]. This antioxidant activity may help preserve dentin, reduce demineralization, and limit host-mediated tissue destruction. In addition, previous studies suggest that α-carotene may modulate immune responses by downregulating pro-inflammatory mediators, supporting soft tissue repair and periodontal stability. It may also inhibit acid production by cariogenic oral bacteria, thereby reducing acidogenic biofilm formation on exposed root surfaces. Finally, as a provitamin A carotenoid, α-carotene contributes to mucosal and epithelial health by promoting keratin formation, immune function, and barrier integrity [48]. These combined actions may explain the observed association between serum α-carotene levels and root caries by stabilizing early lesions, preserving dentin, and maintaining long-term oral health (Figure 1). Although these pathways were not directly assessed, they offer a plausible biological rationale and warrant further exploration.

Notably, while root caries is commonly linked with aging, our analysis revealed that age was not a significant predictor after adjusting for dietary and demographic covariates. This finding suggests that the association between α-carotene and root caries is independent of age. Although root caries is most prevalent and commonly studied in older adults, this age-independent relationship is particularly important given the projected growth of the aging population, and it highlights the potential role of carotenoid-based dietary guidance as a universal preventive strategy that may benefit all individuals, regardless of age. A previous study involving U.S. adults aged 20 and older identified an association between higher serum α-carotene levels and reduced mortality from chronic inflammatory conditions, pointing to broader systemic benefit and underscoring the need to investigate its potential protective effects in oral health [49]. While the biological mechanisms through which carotenoids benefit oral health are well documented, the specific role of α-carotene in preventing root surface decay has not been fully studied.

The observed sociodemographic patterns emphasize the importance of equity in oral health. These findings may reflect disparities in dietary quality, preventive care access, or fluoride exposure [50]. Our results add to the emergent body of evidence advocating for nutrition-driven interventions and a collaborative, multidisciplinary approach to oral health [51,52,53]. As root caries rates continue to rise, identifying protective dietary factors such as α-carotene may inform public health guidelines and enhance preventive care strategies. Promoting the ingestion of α-carotene-rich foods, such as carrots, pumpkins, and leafy greens, or considering supplementation, may serve as a valuable adjunct to standard caries preventive care [27]. Future research should prioritize prospective cohort studies and randomized controlled trials to confirm causality, define optimal intake thresholds, and clarify the biological mechanisms. Interventional studies increasing dietary consumption or supplementation, especially among high-risk groups, may yield stronger clinical evidence. Additional investigation into α-carotene’s influence on oral microbiota, salivary oxidative balance, and immune function could further clarify its mechanistic understanding [54]. Exploring its synergistic effects with other antioxidants, such as vitamin C and polyphenols, may also support the development of comprehensive dietary strategies for oral health [55].

### Limitations of the Research

This study is limited by its cross-sectional design, which does not allow for the establishment of causality and instead identifies α-carotene only as a potential risk indicator. Although the large sample size increases the study’s statistical power and generalizability to the broader U.S. population, it also raises the likelihood of detecting statistically significant associations with small effect sizes that may not be clinically meaningful. To address this, we emphasized effect sizes, confidence intervals, and the practical implications of our findings rather than relying solely on *p*-values. While our findings align with the existing literature and suggest biological plausibility, longitudinal and mechanistic research is necessary to establish temporal and causal relationships. NHANES does not collect data on broader dietary habits, fluoride exposure, lifestyle factors, or genetic variations influencing carotenoid metabolism, which could be important unmeasured confounders in this association. Reliance on a single serum measurement provides only a snapshot of nutritional status and does not capture long-term dietary behaviors or fluctuations in nutrient levels. Additionally, root caries identification in NHANES relied exclusively on visual and tactile assessments without radiographic imaging, which may have led to under- or overestimation of lesion prevalence, especially for early-stage lesions.

## 5. Conclusions

This secondary analysis of 2017–2018 NHANES data identified an inverse association between serum α-carotene levels and the prevalence of root caries in a nationally representative sample of U.S. adults, suggesting a potential protective role for this dietary antioxidant in oral health. These findings provide a foundation for future research to explore the mechanisms by which α-carotene and other antioxidants influence oral health outcomes, potentially expanding their application in clinical practice and public health policy.

## Figures and Tables

**Table 1:** Comparison of population characteristics between individuals with and without root caries.

Covariates		No Root Caries N = 17,701 (92.09%)	Root Caries N = 1520 (7.91%)	Total N = 19,221	*p*-Value
Sex	Male	8586 (48.34%)	861 (54.20%)	9447 (48.86%)	<0.001
	Female	9115 (51.66%)	659 (45.80%)	9774 (51.14%)	
Age (in years)	Less than 6	2980 (8.14%)	0(0.00%)	2980 (7.41%)	<0.0001
	6–11	2447 (8.57%)	0(0.00%)	2447 (7.80%)	
	12–18	2231 (10.36%)	1 (0.027%)	2232 (9.44%)	
	19–44	4355 (33.86%)	445 (34.59%)	4800 (33.93%)	
	45–59	2305 (19.20%)	408 (30.62%)	2713 (20.22%)	
	Above 60	3383 (19.87%)	666 (34.76%)	4049 (21.20%)	
Education	Grade Level 0–11	2025 (11.80%)	453 (21.11%)	2478 (12.92%)	<0.0001
	High School/General Educational Development (GED)	2136 (22.71%)	425 (32.93%)	2561 (23.94%)	
	>High School	5593 (65.48%)	634 (45.96%)	6227 (63.14%)	
Race	Mexican American	5050 (18.33%)	364 (13.05%)	5414 (17.86%)	<0.0001
	White	5692 (59.72%)	523 (61.49%)	6215 (59.88%)	
	Black	3799 (11.45%)	445 (16.39%)	4244 (11.89%)	
	Asian	2105 (5.82%)	104 (3.19%)	2209 (5.58%)	
	Other	1055 (4.68%)	84 (5.88%)	1139 (4.79%)	
FPL	<100% FPL	3752 (15.13%)	399 (24.16%)	4151 (15.93%)	<0.0001
	100–199% FPL	4295 (20.81%)	490 (31.35%)	4785 (21.74%)	
	200–399% FPL	4064 (28.37%)	318 (28.60%)	4382 (28.39%)	
	4000%+ FPL	3499 (35.68%)	123 (15.89%)	3622 (33.94%)	
Treatment For Gum Disease	Yes	1053 (23.37%)	121 (18.14%)	1174 (22.74%)	0.06
	No	3009 (76.63%)	537 (81.86%)	3546 (77.26%)	

FPL: Federal Poverty Level.

**Table 2:** Descriptive summary of continuous variables: comparison with and without root caries.

Continuous Variable	No Root Caries Mean (SD)	Root Caries Mean (SD)	*p*-Value
Serum α-Carotene (μg/dL)	5.17 (9.58)	3.21 (5.52)	<0.0001

**Table 3:** Multiple logistic regression model: association between serum α-carotene levels and root caries in the United States, 2017–2018.

Covariate		Odds Ratio	Confidence Interval		*p*-Value
			Lower	Upper	
α-Carotene		0.91	0.86	0.97	0.004
Age (Reference: 19–44)	45–59	1.17	0.76	1.81	0.45
	Above 60	1.22	0.86	1.73	0.24
Sex (Reference: Females)		0.86	0.64	1.15	0.28
Education Level (Reference: Below Grade 11)	High School/ General Educational Development (GED)	0.70	0.44	1.09	0.107
	>High School	0.80	0.56	1.15	0.208
Race (Reference: Mexican Americans)	White	1.44	0.85	2.44	0.162
	Black	1.77	1.15	2.74	0.013
	Asian	1.06	0.67	1.67	0.806
	Other	1.69	0.76	3.73	0.181
FPL (Reference: <100% FPL)	100–199% FPL	0.69	0.47	1.03	0.066
	138–399% FPL	0.35	0.22	0.55	0.000
	400%+ FPL	0.17	0.08	0.33	0.000
Treatment For Gum Disease		1.25	0.83	1.87	0.259

FPL: Federal Poverty Level.

## Data Availability

The data used in this study is publicly available through the CDC. Additional data supporting the findings can be accessed upon reasonable request by contacting the corresponding author. Any data sharing will comply with privacy regulations and necessary safeguards. For further details, inquiries can be directed to the corresponding author.

## Abbreviations

The following abbreviations are used in this manuscript:

ROS	Reactive Oxygen Species
MMP	Matrix Metalloproteinase
